# Physicochemical Approaches to Medically Relevant Biofilms: Detection, Characterization, Prevention, Disruption, and Magnetic-Field-Based Strategies

**DOI:** 10.3390/antibiotics15100965

**Published:** 2026-09-30

**Authors:** Victor Andrei Drugă, Mărioara Drugă, Maria Șorop-Florea, Răzvan Mihai Șușan, Monica Maria Șușan, Petrinela Ceachir, Monica Sorina Licker, Valentin Laurențiu Ordodi

**Affiliations:** 1 Doctoral School, “Victor Babeș” University of Medicine and Pharmacy, Eftimie Murgu Square No. 2, 300041 Timișoara, Romania; victor.druga@umft.ro (V.A.D.); petrinela.daliu@umft.ro (P.C.); 2 Department of Food Science, Faculty of Food Engineering, University of Life Sciences “King Mihai I” from Timișoara, Calea Aradului 119, 300645 Timișoara, Romania; marioaradruga@usvt.ro; 3 Department of Health and Human Motricity, Faculty of Medical and Behavioral Sciences, “Constantin Brâncuși” University, Tineretului Street No. 4, 210185 Târgu Jiu, Romania; 4 Department of Family Medicine, Centre for Preventive Medicine, “Victor Babeș” University of Medicine and Pharmacy, Eftimie Murgu Square No. 2, 300041 Timișoara, Romania; razvansusan@umft.ro; 5 Department of Internal Medicine I, Discipline of Medical Semiology I, Centre for Preventive Medicine, “Victor Babeș” University of Medicine and Pharmacy, Eftimie Murgu Square No. 2, 300041 Timișoara, Romania; susan.monica@umft.ro; 6 Multidisciplinary Research Center on Antimicrobial Resistance (MULTI-REZ), “Victor Babeș” University of Medicine and Pharmacy, Eftimie Murgu Square No. 2, 300041 Timișoara, Romania; licker.monica@umft.ro; 7 Microbiology Laboratory, “Pius Brînzeu” County Clinical Emergency Hospital, 300723 Timișoara, Romania; 8 Microbiology Department, “Victor Babeș” University of Medicine and Pharmacy, Eftimie Murgu Square No. 2, 300041 Timișoara, Romania; 9 Department of Applied Chemistry, Organic and Natural Compounds Engineering (CAICON), Faculty of Chemical Engineering, Biotechnologies and Environmental Protection, Politehnica University Timișoara, Vasile Pârvan Blvd. No. 6, 300223 Timișoara, Romania; valentin.ordodi@upt.ro; 10 Immuno-Physiology and Biotechnologies Center, Department of Functional Sciences, “Victor Babeș” University of Medicine and Pharmacy, Eftimie Murgu Square No. 2, 300041 Timișoara, Romania; 11 Center for Gene and Cellular Therapies in the Treatment of Cancer—OncoGen, “Pius Brînzeu” County Clinical Emergency Hospital, 156 Liviu Rebreanu Blvd., 300723 Timișoara, Romania

**Keywords:** biofilm, physicochemical methods, magnetic field, electrochemical impedance spectroscopy, biofilm monitoring, ultrasound, antimicrobial photodynamic therapy, cold atmospheric plasma, nanomaterials, surface modification

## Abstract

**Background:** Medically relevant biofilms are difficult to detect, characterize, and eradicate because their behavior depends on microbial species, biofilm maturity, extracellular matrix, surface properties, and environmental conditions. Physicochemical approaches provide complementary strategies for monitoring biofilm development and for preventing or disrupting established communities. This structured narrative review examines electrical and electrochemical methods, ultrasound, light-based and photodynamic treatments, plasma and thermal approaches, nanomaterials and photocatalysis, surface modification, and physicochemical detection methods, with particular emphasis on magnetic-field-based strategies. Contemporary primary studies published from January 2025 to July 2026 were considered together with selected earlier studies providing methodological or comparative context. **Findings:** Physicochemical approaches produced substantial effects across bacterial and fungal biofilm models, but outcomes were strongly dependent on experimental conditions and assessment methods. Cultivability, biomass, metabolic activity, adhesion, detachment, architecture, and physicochemical signals represent distinct aspects of biofilm behavior and should not be interpreted interchangeably. Real-time monitoring methods provide valuable temporal information but require validation against biological endpoints. Magnetic approaches particularly illustrate this methodological heterogeneity. Direct magnetic-field exposure produced variable and incompletely standardized responses, whereas magnetothermal, magnetomechanical, and magnetically guided systems generally showed more consistently quantified antibiofilm effects. Further progress requires standardized physicochemical characterization, appropriate mechanistic controls, clinically representative models, and complementary assessment of biofilm viability, biomass, structure, and regrowth.

## 1. Introduction

Medically relevant biofilms contribute to persistent and difficult-to-treat infections on host-associated surfaces, medical devices, implants, and other biomaterials. Following adhesion, bacterial and fungal microorganisms can develop structured communities embedded within an extracellular polymeric matrix, increasing tolerance to antimicrobial treatment, host defenses, and mechanical clearance [1,2].

Experimental investigation of biofilms requires complementary methods for detection, quantification, and characterization because viability, biomass, metabolic activity, matrix composition, and architecture represent distinct biological properties. Culture-based, staining, metabolic, microscopic, molecular, and physicochemical methods therefore provide different and not necessarily interchangeable measures of biofilm development or treatment response [3,4].

A wide range of physicochemical approaches has been investigated for preventing adhesion, modifying biofilm development, disrupting established biofilms, or monitoring their behavior. These include electrical and electrochemical methods, ultrasound, light-based and photodynamic treatments, plasma and thermal approaches, nanomaterials and photocatalytic systems, surface modification, and magnetic or electromagnetic fields [5,6]. Their apparent efficacy depends not only on the intervention itself but also on exposure conditions, the experimental model, and the biological endpoint used for assessment [6,7,8].

Magnetic and electromagnetic approaches are of particular interest because their reported effects on microbial growth and biofilm formation vary considerably with field type, intensity, frequency, exposure duration, microorganism, biofilm model, and assessment method [6,7]. In addition, direct magnetic-field exposure, magnetothermal treatment, magnetomechanical actuation, and magnetically guided systems represent mechanistically distinct strategies that should not be interpreted as equivalent. This methodological heterogeneity may contribute to the variable and sometimes contradictory findings reported across studies [7,8].

An overview of the principal physicochemical approaches, their applications, assessment endpoints, and major methodological considerations is provided in Table 1.

The primary aim of this structured narrative review is to provide an overview of major physicochemical approaches applied to medically relevant biofilms, including strategies for sensing, characterization, prevention, and treatment, while summarizing the quantitative and semi-quantitative methods used to assess their effects. A complementary aim is to provide a common interpretive framework for comparing these heterogeneous approaches by distinguishing the biological endpoints used to evaluate biofilm response and by critically examining experimental models, exposure conditions, proposed mechanisms, and methodological limitations. Particular emphasis is placed on magnetic- and electromagnetic-field-based strategies, with direct magnetic-field effects considered separately from magnetothermal, magnetomechanical, and magnetically guided approaches.

## 2. Physicochemical Detection, Characterization, and Real-Time Monitoring

Physicochemical detection methods enable repeated or continuous assessment of biofilm development without relying exclusively on destructive endpoint assays. Electrochemical impedance spectroscopy (EIS) is among the most extensively investigated approaches, allowing longitudinal monitoring of changes at the biofilm–electrode interface during adhesion, maturation, treatment, and regrowth [9,10,11,12,13,14,15]. Complementary techniques such as quartz crystal microbalance with dissipation monitoring (QCM-D), optical coherence tomography (OCT), and label-free optical imaging provide information on hydrated mass, viscoelasticity, architecture, and metabolic state [16,17,18].

EIS platforms can detect biofilm-associated changes at early developmental stages. Riordan et al. identified measurable *Pseudomonas aeruginosa* impedance changes approximately 1 h after inoculation, with established biofilm producing an approximately 40% decrease in impedance modulus relative to medium-only controls [9]. Microfluidic platforms have similarly enabled continuous monitoring of *Staphylococcus aureus* and *Candida albicans* biofilms [11,12]. Impedance-based systems have also been used to follow biofilm development, treatment response, and subsequent regrowth across *S. aureus*, *Escherichia coli*, *Salmonella*, and *P. aeruginosa* models [15,19,20].

Impedance is not, however, a universal surrogate for biofilm biomass or viability. Staphylococcal and *E. coli* biofilms may increase charge-transfer resistance as cells and extracellular matrix accumulate [11,13,14], whereas electroactive biofilms can produce decreases in low-frequency impedance through redox-mediated extracellular electron-transfer processes [9,14]. Signal magnitude and direction therefore depend on microbial physiology, biofilm developmental stage, electrode configuration, medium composition, and interrogation frequency. EIS is consequently better regarded as a composite physicochemical signature of the biofilm–electrode system than as a directly transferable measure of viable biofilm burden. This interaction between electrical measurement and intervention is considered further in Section 3.

Other longitudinal methods quantify complementary dimensions of biofilm development. QCM-D detects changes in hydrated mass and viscoelastic behavior during microbial attachment and matrix maturation [16], whereas OCT provides non-contact measurements of thickness, roughness, porosity, and internal architecture [17]. Label-free optical metabolic imaging can additionally resolve spatial heterogeneity in microbial metabolic state [18]. These techniques are complementary rather than interchangeable: a major advantage is their ability to provide temporal or spatially resolved information, while a shared limitation is that their physicochemical signals require biological interpretation. Electrical, mechanical, structural, and metabolic measurements remain platform- and condition-dependent and should therefore be validated against appropriate microbiological or biological endpoints [21,22].

As the studies reviewed use heterogeneous biological and physicochemical endpoints, their interpretation requires consideration of what each measurement represents and what it cannot establish independently. The principal assessment endpoints and their interpretive limitations are summarized in Table 2.

## 3. Electrical and Electrochemical Approaches

Electrical and electrochemical approaches can be used both to characterize biofilm-associated interfacial properties and to modify or disrupt biofilms. Foundational work established this distinction between electrical measurement and intervention. The bioelectric effect was described in early studies showing enhanced antibiotic activity at current densities below approximately 100 μA/cm^2^ [8,23,24], while electrochemical treatment was subsequently shown to reduce *Staphylococcus epidermidis* through electrolytic generation of hypochlorous acid [25]. Analytical approaches, including EIS, voltammetry, ζ-potential, electrophoretic mobility, and dielectric measurements, provide complementary information on biofilm-associated electrical properties. EIS permits longitudinal assessment of the biofilm–electrode interface, although its output represents a composite physicochemical signal rather than a direct measure of viable biomass [26,27,28].

Electrical interrogation may itself influence biofilm behavior. Balato et al. exposed mature 96 h MRSA biofilms to impedance measurements over 1 Hz–10 MHz and observed reductions in biomass and cultivability, with prominent effects at approximately 10–100 kHz [29]. Subsequent electric-field exposure also produced a transient increase in vancomycin susceptibility [30]. These findings demonstrate that electrical measurement cannot always be assumed to be biologically passive and that frequency, amplitude, and cumulative exposure may influence biofilm response.

Electrochemical intervention may also operate through the generation of antimicrobial compounds. Al-Qurahi et al. operated an electrochemical catheter hub at 1.5 V versus Ag/AgCl for 3 h, generating hypochlorous acid and producing a mean 4.40 ± 0.05 log_10_ CFU/mL reduction in *Acinetobacter baumannii* biofilm [31]. Electrical and electrochemical approaches may therefore act through distinct mechanisms, including direct field effects, altered antimicrobial susceptibility, changes at the biofilm–electrode interface, and electrochemically generated antimicrobial species. Electroactive materials provide another strategy: Levin et al. developed a conductive bacterial cellulose–carbon nanotube composite dressing that enhanced vancomycin activity against *Staphylococcus aureus*, with one hour of combined electrical and antibiotic treatment reducing subsequent biofilm-forming capacity by nearly 92% [32].

Contemporary electrical and electrochemical studies further illustrate the diversity of analytical and interventional configurations. Grassi et al. used electrochemical impedance spectroscopy with sputtered iridium oxide electrodes to detect biofilm growth within 24 h and distinguish *Staphylococcus aureus* from *Pseudomonas aeruginosa* biofilms [33]. Bernaus et al. evaluated pulsed electrical fields against *Staphylococcus epidermidis* biofilms on cobalt–chrome implant surfaces, reporting the greatest removal when electrical treatment was combined with high-pressure pulse lavage [34]. O’Connor et al. combined electrochemical sensing with microscopy on carbonized porous silicon substrates, reporting *S. epidermidis* surface coverage of approximately 59.3–86.7% after 24 h and nearly 99% by 72 h on selected substrates [35]. Kulshrestha and Gupta similarly used an SPE-enabled microfluidic EIS platform for dynamic assessment of *S. aureus*, *Candida albicans*, and polymicrobial biofilms during antibiofilm treatment [36]. These systems reinforce the need to interpret electrochemical signals in relation to independently measured biological endpoints.

Electrokinetic measurements provide complementary information on microbial surface properties. Lavoie et al. measured ζ-potential at 25 °C in staphylococcal isolates grown in supplemented tryptic soy broth containing 1.25% dextrose, 25 mg/L calcium, and 12.5 mg/L magnesium; weak biofilm-forming isolates exhibited significantly more negative ζ-potentials than strong biofilm formers across the assessed stages [37]. In *Candida albicans*, Johnson et al. measured cells suspended in 248 mM sucrose, 16.7 mM dextrose, 250 μM MgCl_2_, and 100 μM CaCl_2_ at measured conductivities of 43 and 150 mS/m. The strongly biofilm-forming *nrg1Δ/Δ* strain showed less negative ζ-potentials (−14.8 ± 0.6 and −16.9 ± 0.4 mV) than the wild type (−18.2 ± 2.8 and −19.8 ± 1.4 mV), respectively [38]. Together, these findings associate less negative surface charge with stronger biofilm-forming behavior. However, not all physicochemical measurement conditions requested for direct comparison, including pH and ionic strength, were reported in the original studies. Such measurements primarily characterize surface charge and associated biofilm-forming behavior; their interpretation should therefore follow the endpoint-specific considerations summarized in Table 2.

## 4. Ultrasound and Acoustic Methods

Ultrasound is used in biofilm research primarily as an intervention evaluated by independent biological methods or as a preanalytical technique for detaching microorganisms from surfaces. Foundational studies showed that low-intensity ultrasound could enhance antimicrobial activity without necessarily removing the biofilm: Qian et al. reported nearly 2-log greater killing of *Pseudomonas aeruginosa* biofilms with 500 kHz, 10 mW/cm^2^ ultrasound combined with gentamicin [39], while subsequent work demonstrated frequency-dependent enhancement at 70 kHz–10 MHz, with lower frequencies producing greater effects [40]. In diagnostic applications, Trampuz et al. reported a sensitivity of 78.5% for sonicate-fluid culture versus 60.8% for tissue culture, with comparable specificity [41]. Ultrasound outcomes nevertheless depend strongly on frequency, intensity, duty cycle, cavitation, exposure duration, biofilm maturity, and processing conditions [42,43].

Recent acoustic-intervention studies demonstrate substantial but mechanism-dependent effects. Ultrasound-triggered antimicrobial nanodroplets reduced the minimum biofilm eradication concentration by approximately 44-fold compared with free drug [44]. Using a 2 MHz focused acoustic vortex at 1.75 MPa, 10% duty cycle, and 180 s exposure, Li et al. achieved approximately 97% removal of *Escherichia coli* biofilm, compared with 7% using conventional focused ultrasound under matched conditions [45].

Batchelor et al. similarly reported 94 ± 2% removal of *Staphylococcus aureus* biofilm with high-pressure ultrasound and microbubbles, compared with 16 ± 13% using ultrasound alone [46]. Vancomycin-loaded microbubbles improved disruption and killing of MRSA biofilms [47], while ultrasound-triggered voriconazole delivery reduced *Candida albicans* biomass by approximately 63–73% and viability by 66–84%, depending on the surface [48]. In an orthopedic implant model, sonication brushing reduced viable *Staphylococcus aureus* by approximately 87% and retained biofilm biomass by 78%; combining brushing with pulsatile lavage increased these reductions to approximately 99% and 86%, respectively [49]. These results emphasize that detachment, killing, enhanced drug penetration, and loss of surface-associated biomass are distinct outcomes.

As a preanalytical method, sonication is instead used to release biofilm-associated microorganisms from surfaces for subsequent microbiological recovery. In an in vitro prosthetic-joint-infection model, Silva et al. examined staphylococcal and Gram-negative biofilms of different maturities and showed that sonication disrupted biofilm structure while releasing cultivable bacteria from the surface [50]. Schweizer et al. further showed that adding 0.001% saponin increased recovery by up to 2.2 log_10_ CFU for *Staphylococcus epidermidis* [51]. In a clinical comparison of 415 patients, direct sonication achieved 88% sensitivity versus 69% for conventional sonication, although specificity was lower (84% vs. 93%) [52]. Trallero-Calvo et al., analyzing 457 sonicated implants, reported a median bacterial burden of approximately 40,000 CFU/mL, illustrating the quantitative range obtainable within a defined protocol [53]. Because sonication procedures remain heterogeneous in acoustic parameters, processing protocols, culture methods, and diagnostic thresholds [54], sonication-derived CFU should be interpreted as protocol-dependent recovery of cultivable organisms rather than a direct estimate of total biofilm burden, consistent with the endpoint-specific considerations summarized in Table 2.

## 5. Light-Based and Photodynamic Methods

Light-based approaches include antimicrobial blue light, antimicrobial photodynamic therapy (aPDT), photoactive surfaces, and photothermal or nanoparticle-assisted systems. Their effects on biofilm viability, biomass, metabolic activity, and structure depend on wavelength, radiant exposure, photosensitizer chemistry, microorganism, biofilm maturity, surface properties, and the selected endpoint. Earlier work established the application of aPDT to biofilm models, in which light activation of a photosensitizer generates singlet oxygen and other reactive oxygen species [55,56], whereas antimicrobial blue light can exert antimicrobial activity without an externally added photosensitizer [57].

Negri et al. reported that antimicrobial blue light reduced recoverable organisms in wound-associated bacterial biofilms and increased susceptibility to tetracycline-class antibiotics [57]. In *Candida albicans*, probiotic-enhanced aPDT reduced both biofilm formation and established biofilm burden [58]. Using a different photosensitizer strategy, Amorim et al. treated biofilms formed by clinical isolates from infected diabetic foot ulcers with a dicationic chlorin and 660 nm light, producing reductions exceeding 3 log_10_ CFU across tested biofilms; the addition of potassium iodide further enhanced activity and resulted in eradication below the detection limit in several cases [59]. Curcumin-mediated aPDT also showed a strong dependence on biofilm maturity, reducing developing *Candida* biofilms by approximately 68–87%, whereas reductions in mature biofilms were smaller and not statistically significant [60].

More complex light-responsive systems showed enhanced but incomplete activity. A near-infrared-triggered platform combining gold nanostars, mesoporous silica, photothermal curcumin release, and photodynamic activation reduced *Staphylococcus aureus* and *Pseudomonas aeruginosa* biofilms by approximately 2.16 and 1.77 log_10_ CFU, respectively [61]. Viable organisms nevertheless remained. Tetracationic near-infrared porphyrins also showed structure-dependent activity against mature *S. aureus* biofilms [62]; notably, the compound with the greatest planktonic phototoxicity was not the most effective against biofilm, indicating that physicochemical properties, matrix interactions, and penetration can substantially influence antibiofilm efficacy.

Photoactive materials provide an additional strategy by generating reactive oxygen species at the material–biofilm interface. Phloxine B-functionalized polyurethane produced light-dependent reductions in mixed *Candida albicans–S. aureus* biofilms [63], while photoactivated silver nanoparticles containing *Moringa oleifera*-derived compounds reduced *C. albicans* biofilm formation [64]. Porphyrin-loaded nanoparticles combined with potassium iodide also decreased metabolic activity in *C. albicans* and *Pichia kudriavzevii* biofilms [65]. In such multimodal systems, however, the contributions of photodynamic activity, metal toxicity, chemical enhancers, and associated compounds may be difficult to separate.

Clinically representative models showed treatment- and species-dependent responses. In diabetic-foot-ulcer-like mono- and polymicrobial biofilms, methylene-blue aPDT alone showed limited activity at lower photosensitizer concentrations, whereas sequential combination with antibiotics substantially enhanced killing of *Pseudomonas aeruginosa*, and *Enterococcus faecalis* in the mixed biofilm remained comparatively less affected [66]. By contrast, methylene-blue aPDT produced reductions exceeding 3 log_10_ CFU in polymicrobial endotracheal-tube biofilms [55]. Activity against *S. aureus* and *Escherichia coli* biofilms on polyethylene, titanium alloy, cobalt–chromium–molybdenum alloy, and polymethyl methacrylate also varied with microorganism, material, biofilm maturity, photosensitizer concentration, and radiant exposure [56].

Overall, developing biofilms generally appeared more susceptible than mature or complex biofilm models [58,60,64,65]. Complete eradication remained uncommon, although selected combinations, such as chlorin-mediated aPDT potentiated with potassium iodide, achieved culture-negative outcomes in some biofilms [59]. Interpretation of these findings should account for the specific biological endpoint assessed, as summarized in Table 2.

## 6. Plasma and Thermal Methods

Plasma- and heat-based interventions are used to prevent biofilm formation or reduce established communities. Cold atmospheric plasma can generate reactive oxygen and nitrogen species, charged particles, electric fields, ultraviolet radiation, and limited heating, with their relative contributions depending on source configuration and whether exposure is direct or indirect [67,68]. Thermal approaches instead use conductive, thermoelectric, or induction-mediated heating, with efficacy determined by temperature, exposure duration, heating kinetics, spatial heat distribution, and the surrounding environment [69,70].

In situ plasma-activated water treatment of 48-h *Escherichia coli* UTI89 and *Staphylococcus aureus* NCTC8325 biofilms reduced viable counts by 6.76 ± 0.01 and 6.82 ± 0.02 log_10_ CFU/mL, respectively, relative to the initial cultivable burden, reaching the detection limit after 13 and 11 min of treatment [71].

Surviving *E. coli* nevertheless exhibited oxidative-stress and repair responses, indicating that major loss of cultivability may coexist with persistent cellular responses [72]. Direct plasma also reduced imipenem-resistant *Klebsiella pneumoniae* biofilms [73], while comparison of three plasma devices against *S. aureus* demonstrated several-log reductions that varied with source design, plasma chemistry, temperature, and exposure duration [74].

In a 72 h endoscope-channel model, non-thermal plasma reduced *Pseudomonas aeruginosa*, *S. aureus*, *E. coli*, and *K. pneumoniae* biofilms by approximately 4.98–7.49 log_10_ CFU/cm^2^, with no recoverable organisms under selected conditions [75]. Plasma-assisted functionalization of urinary catheters also reduced colonization by methicillin-resistant *S. aureus* and multidrug-resistant *P. aeruginosa*, representing prevention of biofilm development rather than treatment of an established biofilm [76]. Similarly, gliding-arc plasma applied to 48 h *Candida albicans* biofilms produced conditions with no recoverable growth while reducing respiratory activity, membrane integrity, hyphal structure, and attachment [77]. Plasma exposure also altered MRSA biofilm architecture, stress responses, and susceptibility to subsequent plasma or mupirocin treatment [78]. Together, these findings show that culture negativity, metabolic inhibition, structural disruption, and prevention of recolonization represent related but distinct outcomes.

Thermal approaches may combine microbial killing with biofilm disruption or detachment. In a mature implant-biofilm model, induction heating of seven-day *Staphylococcus aureus* biofilms at 60 °C for 1 min produced an approximately 7.3-log_10_ reduction in viable bacteria [69]. Such experimental thermal exposures must also be interpreted in relation to host-tissue tolerance, as achieving bactericidal temperatures at the implant surface may expose adjacent bone and soft tissues to potentially damaging thermal doses [69,70]. More recent thermoelectric heating experiments showed that *P. aeruginosa* biofilms can disperse from heated surfaces, with surrounding fluid reducing treatment efficacy and providing a reservoir for released organisms [79]. In an orthopedic implant model, induction heating combined with antibiotics or synthetic antimicrobial and antibiofilm peptide SAAP-148 reduced *S. aureus* viability more effectively than the individual treatments; analysis of both the implant and surrounding fluid indicated that the effect was not explained solely by redistribution [80].

Interpretation should account for the endpoint-specific distinctions summarized in Table 2. Plasma studies should report source configuration, electrical and gas parameters, temperature, and relevant reactive-species characteristics [68,74], while thermal studies should characterize treatment temperature, heating kinetics, duration, spatial distribution, and the relationship between surface and bulk temperatures [69,70,79]. Where treatment may disperse rather than eradicate biofilm, assessment should include both surface-associated and released populations using complementary endpoints.

## 7. Nanomaterial-Based and Photocatalytic Methods

Nanomaterial-based physicochemical approaches are used to prevent microbial attachment, inhibit biofilm development, or reduce established biofilms. Investigated systems include metallic and metal-oxide nanoparticles, selenium-based materials, carbon composites, nanomaterial-enabled device coatings, antimicrobial conjugates, and photothermal or photocatalytic systems. Their activity depends on physicochemical properties, including particle size, surface charge, morphology, aggregation, dissolution, and matrix penetration [81,82]. Earlier studies demonstrated inhibition of biofilm formation by silver and ZnO nanoparticles against organisms including *Pseudomonas aeruginosa* and *Staphylococcus epidermidis*, while ZnO also reduced *P. aeruginosa* pyocyanin production and quorum-sensing-associated phenotypes [83,84,85], suggesting effects beyond direct microbial killing.

Preventive applications include ZnO nanoparticle coatings that reduced bacterial attachment to urinary catheters [86], polyhydroxy-fullerene-based metal nanoparticle coatings that decreased *Escherichia coli* biofilm formation on polyurethane [87], and biogenic selenium nanoparticle coatings that inhibited *Candidozyma auris* colonization on catheter material [88]. These approaches predominantly target attachment and early biofilm development rather than eradication of mature communities. In an in vitro bladder model, curcumin-modified silver nanoparticle-coated catheters showed approximately 70–80% lower crystal violet biomass and 60–80% lower MTT metabolic activity than uncoated catheters during biofilm maturation and catheter blockage [89]. Prevention of crystalline biofilm and catheter encrustation nevertheless represents a distinct outcome from microbial killing.

Quantitative efficacy varied considerably among formulations. A graphene oxide–silver composite at 0.25 mg/mL reduced crystal violet biomass by approximately 66% for *P. aeruginosa* and 57% for *Candida albicans* [90]. Biogenic zinc nanoparticles inhibited biofilm formation by up to approximately 80% for *Staphylococcus aureus* and around 50% for *E. coli* and yeast species [91], whereas ZnO incorporated into an eggshell-derived matrix produced only approximately 30% inhibition against *P. aeruginosa* [92]. Baicalin-coated ZnO nanoparticles produced approximately 80–94% inhibition of *S. aureus* biofilm formation [93]. Biosynthesized selenium nanoparticles also showed activity against carbapenem-resistant *P. aeruginosa*: combination with imipenem produced antibacterial synergy with FICI values of 0.37–0.50, while selenium nanoparticle treatment alone or in combination significantly reduced biofilm formation and expression of the biofilm-associated *Bap* and *ompA* genes [94]. Physicochemical formulation itself can substantially alter efficacy; chemically synthesized ZnO nanoparticles reduced *C. auris* adhesion by 67.9 ± 2.35% at 150 µg/mL, whereas a biologically synthesized formulation showed negligible inhibition, with differences also observed in particle size, ζ-potential, and adhesin-gene expression [95]. These findings illustrate that nanoparticle-mediated activity cannot be attributed to composition alone but also depends on synthesis-dependent physicochemical properties and interactions with the biofilm.

Photocatalytic nanomaterials represent a related light-responsive strategy. Upon irradiation, materials such as TiO_2_ can generate reactive species, including hydroxyl radicals, superoxide, and hydrogen peroxide, producing oxidative damage at the material–biofilm interface [96]. Doping and composite formation can modify optical and surface properties and potentially extend photocatalytic activity toward the visible spectrum [96]. Because such systems may combine photocatalysis with intrinsic material toxicity, surface effects, or other antimicrobial mechanisms, the dominant contribution should be distinguished experimentally.

Nanomaterial studies also present specific analytical limitations. Crystal violet measurements reflect retained biomass without distinguishing viable cells, dead cells, and extracellular matrix [90,91,92,93], while metabolic and fluorescence assays may be affected directly by particle absorption, dye binding, reagent reduction, or fluorescence quenching. Material-only blanks and assay-interference controls are therefore important. Quantitative interpretation should additionally include physicochemical characterization of particle size, morphology, surface charge, aggregation state, and, where relevant, dissolution or ion release. Earlier photocatalytic work has likewise emphasized the need for appropriate light, dark, and catalyst-only controls [96]. These findings should be interpreted according to the endpoint-specific considerations summarized in Table 2.

## 8. Surface Modification and Antifouling Strategies

Surface modification is primarily a preventive physicochemical strategy that alters the material–microorganism interface to limit initial adhesion and subsequent biofilm development. Relevant properties include surface chemistry, wettability, charge, roughness, topography, and porosity. Approaches can be broadly divided into passive antifouling interfaces that reduce attachment without direct killing, active surfaces incorporating contact-active or releasable antimicrobial agents, and engineered micro- or nanotopographies [97,98,99,100,101,102,103,104,105,106,107,108,109,110].

Hydrophilic, PEG-based, and zwitterionic interfaces can markedly reduce nonspecific microbial adhesion [97,98,103,106,107]. A superhydrophilic catheter coating with a water-contact angle of <4°, compared with >90° for untreated silicone, reduced biofilm formation by up to 99% under static conditions and approximately 83% in a five-day flow model [98]. PEG-linked immobilized α-defensin-5 produced approximately 50–99% reductions against *Pseudomonas aeruginosa*, MRSA, and MRSE [97], while a polysulfobetaine coating combined with antimicrobial nanoformulations reduced catheter biofilm by approximately 70% after seven days [103]. Dual antibacterial/antiadhesive polyurethane also retained activity after conditioning with proteins, plasma, and lipids [106], highlighting the importance of evaluating modified surfaces under biologically relevant conditions.

Active surfaces can additionally provide contact-mediated or sustained antimicrobial effects. Chlorhexidine-containing catheter coatings produced an approximately 6-log reduction in biofilm adhesion during prolonged artificial-urine exposure [99]. Porous rifampicin-loaded coatings maintained antimicrobial release for up to 28 days, reducing *P. aeruginosa* adhesion by >99.99% and eight-day biofilm formation by approximately 92.39% [104]. Chitosan-grafted polycaprolactone nanofibers also inhibited *Staphylococcus epidermidis* biofilm formation [101], while silver-treated sutures reduced colonization and biofilm development by *S. aureus* and *P. aeruginosa* [105]. Such systems remain dependent on coating stability, antimicrobial availability, and, for releasable agents, reservoir depletion and release kinetics; prolonged subinhibitory exposure may therefore represent a potential limitation [99,104].

Surface architecture can also influence microbial attachment in a scale- and geometry-dependent manner. Macrotexturing may promote colonization by increasing available surface area and creating protected niches [100], whereas engineered microtopographies can restrict bacterial attachment [108]. Nanoscale architectures may additionally combine topographical effects with contact-mediated antimicrobial activity. After 12 h, copper-coated carbon-infiltrated carbon nanotube surfaces produced a 6.3-log reduction in recoverable *S. aureus* and a 6.9-log reduction in adherent *Pseudomonas aeruginosa*, quantified as CFU/sample relative to bare titanium controls (*p* = 6.1 × 10^−6^ and *p* < 5 × 10^−9^, respectively) [102]. Consequently, roughness parameters such as Ra alone are insufficient to predict biological behavior; feature size, spacing, curvature, surface chemistry, and dimensions relative to microbial cells are also relevant [100,102,108].

Overall, the available evidence predominantly concerns prevention of attachment and early biofilm development rather than eradication of mature biofilms [97,98,99,100,101,102,103,104,105,106]. Apparent efficacy should therefore be interpreted according to the specific endpoint assessed, as summarized in Table 2. Interpretation also requires parallel physicochemical characterization of the modified material, particularly its wettability, surface charge, topography, coating stability, and, where applicable, antimicrobial release kinetics.

## 9. Magnetic Field-Based Approaches

**Figure 1:**
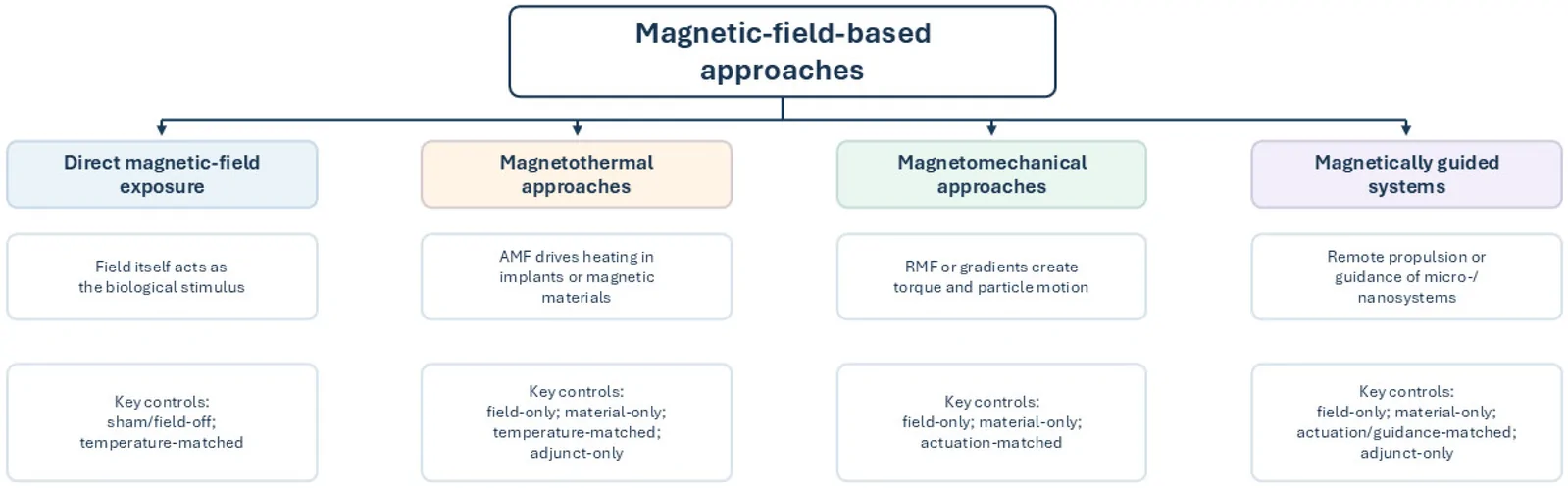
Magnetic-field-based strategies investigated against medically relevant biofilms. AMF: alternating magnetic field; RMF: rotating magnetic field.

Magnetic-field-based approaches comprise mechanistically distinct strategies, including direct magnetic-field exposure, alternating magnetic field (AMF)-mediated heating, rotating magnetic field (RMF)-driven mechanical actuation, and magnetically guided micro- or nanorobotic systems [111,112,113,114,115,116,117,118,119,120]. Importantly, these approaches should not be interpreted as biologically equivalent: in direct-exposure experiments, the magnetic field itself constitutes the principal stimulus, whereas in magnetothermal, magnetomechanical, and robotic systems, it primarily actuates a responsive material or structure. The mechanistic distinctions among the principal magnetic-field-based approaches and the controls required for interpretation are summarized in Figure 1.

Direct magnetobiological effects remain the least standardized and may be inhibitory, neutral, or stimulatory depending on the microorganism and exposure conditions. RMF exposure of approximately 25–34 mT at 5–50 Hz for 60 min produced species-dependent changes in metabolic and biofilm-related activity [121]. Hanini et al. reported increased *Pseudomonas aeruginosa* biofilm formation under a 200 mT static magnetic field, accompanied by changes in biofilm-associated phenotypes [122]. Junka et al. further showed that rotating magnetic fields at 10–50 Hz could enhance the activity of antibiotics and antiseptics against *S. aureus* and *P. aeruginosa* wound biofilms, with effects varying according to field frequency and the measured endpoint [123]. A subsequent study using 5 and 50 Hz RMF found that RMF or octenidine alone did not destroy established biofilms, whereas their combination affected microbial viability, biofilm matrix properties, and antiseptic penetration [124].

In a polymicrobial chronic-wound model containing *Staphylococcus aureus*, *P. aeruginosa*, and *Candida albicans*, Di Fermo et al. found that low-intensity complex magnetic fields, alone or combined with methylglyoxal and red light, reduced cultivable biofilm burden and altered biofilm architecture [114]. Proposed mechanisms involving membrane and surface properties, oxidative balance, ion transport, motility, extracellular polymeric substance regulation, metabolism, and cellular signaling remain incompletely established [121,122,123,125]. These divergent findings emphasize the importance of magnetic dosimetry and standardized biological endpoints when assessing direct field effects.

AMF combined with linezolid produced a mean 5.72-log reduction in Gram-positive biofilm burden, whereas AMF combined with ciprofloxacin produced a mean 5.11-log reduction in Gram-negative biofilms. Viable burden was quantified as log_10_ CFU/cm^2^ on metal rings, and combination treatment produced greater reductions than antibiotic monotherapy and untreated controls [115]. These findings are consistent with earlier evidence that magnetic nanoparticle hyperthermia can enhance antibiotic activity against *S. aureus* biofilms [126]. AMF exposure of *S. aureus* biofilms on titanium also produced substantial biofilm effects despite negligible bulk heating, indicating that bulk temperature alone may not adequately describe the local conditions generated at the implant surface [113]. Associated injury to osteoblast-like cells, however, emphasizes the need to define a therapeutic window between antibiofilm efficacy and host-tissue damage [113,126].

Magnetomechanical approaches convert magnetic torque or particle movement into matrix disruption and cellular injury. RMF-actuated magnetic nanochains increased methicillin susceptibility in resistant *S. epidermidis* biofilms and, when combined with antibiotic treatment, achieved approximately 99.99% killing [111]. Fe_3_O_4_@PDA nanospears similarly produced geometry-dependent disruption that synergized with photothermal treatment [112]. Larger magnetic microrods caused greater *Enterococcus faecalis* biofilm detachment, whereas smaller nanoscale structures produced proportionally greater cellular injury [116]. These findings illustrate that mechanical removal and microbial killing are distinct outcomes and should be evaluated through separate measurements of residual biomass, detached organisms, viability, and matrix disruption.

Magnetic fields can also provide remote guidance and actuation of micro- and nanoscale systems. Fe_3_O_4_ microrobots removed established *S. aureus* and *Escherichia coli* biofilms from complex medical-device surfaces [117], while magnetically propelled micro-rolls achieved an approximately 97% E. coli killing rate in an infected pancreatic-stent model [118]. Catalytic biohybrid microrobots reduced residual *S. aureus* and *P. aeruginosa* viability to approximately 10–14% on catheter-like surfaces [119], and Fe_3_O_4_-based systems have also been investigated for simultaneous biofilm disruption, antimicrobial activity, and catheter-obstruction clearance [120]. In these applications, the magnetic field primarily provides remotely controlled motion, mechanical actuation, heating, or targeted delivery rather than functioning directly as the antimicrobial agent [125].

Clinical translation of AMF-based approaches is constrained not only by antibiofilm efficacy but also by field tolerability and nonspecific tissue heating. Atkinson–Brezovich-type criteria relate allowable field amplitude to frequency, although these empirical limits depend on factors such as coil geometry, treated volume, tissue conductivity, and exposure configuration and should not be interpreted as universal safety thresholds. Eddy-current heating of surrounding conductive tissues or adjacent metallic structures, limited control of local versus bulk temperature, and injury to host cells may therefore define the practical therapeutic window for magnetothermal approaches [113,126].

Interpretation of magnetic-field studies is limited by substantial methodological heterogeneity. Direct-exposure experiments should report magnetic flux density, frequency, waveform, field geometry or gradient, exposure duration, sample position, and thermal conditions. Magnetothermal studies should distinguish bulk, local, and surface temperatures where possible, while magnetomechanical studies additionally require physicochemical characterization of the magnetic material and its actuation conditions. Appropriate controls should distinguish field-only, material-only, adjunct-only, magnetic-actuation, and temperature-matched heating effects.

Among the purposively selected studies, magnetothermal approaches [113,115,126] and magnetomechanical approaches [111,112,116] more frequently reported substantial quantitative antibiofilm effects, whereas direct magnetic-field responses were more variable and less standardized [114,121,122,123,124]. Given the heterogeneity of experimental models, exposure conditions, and assessment methods, this pattern should not be interpreted as a direct comparison of efficacy across magnetic strategies. Further work requires rigorous magnetic dosimetry and assessment using complementary endpoints consistent with the interpretive framework summarized in Table 2 [125,127,128].

## 10. Materials and Methods

### 10.1. Study Design

This study was conducted as a structured narrative review of physicochemical approaches used in experimental biofilm research, with particular emphasis on magnetic and electromagnetic fields. The literature search was designed to prioritize contemporary evidence while retaining selected earlier publications for historical, methodological, and comparative context.

PubMed/MEDLINE and Scopus were searched for English-language primary studies published from 1 January 2025 to 24 July 2026. Biofilm-related terms were combined with category-specific terms covering electrical and electrochemical approaches, ultrasound and acoustic methods, light-based and photodynamic treatments, plasma and thermal interventions, nanomaterials and photocatalysis, physicochemical surface modification, detection and real-time monitoring methods, and magnetic or electromagnetic fields.

In PubMed/MEDLINE, the core term biofilm [ti] was combined with terms searched primarily in the title and abstract; in Scopus, TITLE (biofilm) was combined with corresponding TITLE-ABS-KEY terms. The category-specific search blocks included: electrical and electrochemical approaches (electric field, electrical stimulation, electric current, bioelectric, electrochemical treatment); ultrasound and acoustic methods (ultrasound, ultrasonic, sonodynamic, acoustic cavitation, focused ultrasound); light-based methods (photodynamic, photothermal, photosensitizer, antimicrobial blue light, ultraviolet, near-infrared); plasma and thermal approaches (cold atmospheric plasma, non-thermal plasma, plasma-activated water, hyperthermia, thermal treatment); nanomaterial and photocatalytic approaches (nanoparticle, nanomaterial, nanocomposite, graphene, metal oxide nanoparticle, photocatalysis); surface modification (surface modification, surface functionalization, surface roughness, surface topography, wettability, surface charge, zeta potential, antifouling); physicochemical detection and monitoring (electrochemical impedance spectroscopy, impedimetric, biosensor, quartz crystal microbalance, optical coherence tomography, microcalorimetry, real-time monitoring, label-free); and magnetic or electromagnetic approaches (magnetic field, static magnetic field, alternating magnetic field, pulsed magnetic field, electromagnetic field, magnetic hyperthermia, magnetoelectric, ELF-EMF, PEMF).

Earlier primary studies were included selectively when they represented foundational developments, established methodological principles or quantitative benchmarks, or provided relevant comparison with contemporary findings. Review articles were used for background and comparison with the broader literature and were not considered part of the contemporary primary-study set.

### 10.2. Study Selection and Data Synthesis

Eligible contemporary studies were primarily in vitro or ex vivo investigations of medically relevant bacterial or fungal biofilms reporting at least one quantitative or semi-quantitative biofilm outcome. For detection and preanalytical methods, clinical diagnostic studies involving explanted medical devices, sonicated materials, or other biofilm-associated clinical specimens were also considered when they provided quantitative measures of microbial recovery, diagnostic performance, or biofilm-related burden. These studies were included as evidence for diagnostic or preanalytical applications rather than for in vivo therapeutic efficacy. Animal studies; dental implant and orthodontic studies; planktonic-only investigations; studies on non-medical, environmental, or industrial biofilms; non-primary publications; duplicate records; and studies without a relevant quantitative or semi-quantitative biofilm assessment were excluded. The exclusion of dental and orthodontic studies was intended to maintain the predefined focus on non-dental, medically relevant biofilm models and physicochemical interventions.

As the purpose was a focused narrative synthesis rather than an exhaustive systematic review, 10 representative studies were purposively selected for each physicochemical category. This limit was used to maintain balanced coverage across categories and to avoid disproportionately expanding sections in areas with a substantially larger body of research. Selection emphasized methodological relevance, clarity of experimental reporting, diversity of microorganisms and biofilm models, distinct technologies or exposure conditions, clinically relevant applications, and the availability of quantitative or semi-quantitative biofilm outcomes. Selection was not based on the direction or magnitude of the reported effect. Accordingly, comparisons between physicochemical approaches in this review apply to the purposively selected study set and should not be interpreted as representing the literature as a whole. Magnetic-field studies received particular emphasis.

Extracted information included the microorganism and biofilm model, physicochemical approach, principal exposure or material parameters, assessment methods, and major quantitative or semi-quantitative findings. Results were synthesized narratively within each thematic category. The representative studies and their principal experimental characteristics are summarized in Supplementary Table S1, with additional magnetic exposure and material parameters reported for magnetic-field-based studies where available.

### 10.3. Methodological Considerations

Because of the substantial heterogeneity of experimental models, interventions, and outcome measures, no formal risk-of-bias assessment or meta-analysis was undertaken. Methodological limitations were instead considered narratively, with attention to experimental controls, characterization of exposure conditions or materials, biofilm maturity, choice of assessment method, and agreement between complementary endpoints. Where quantitative effects are reported, the endpoint or assay, comparator, measurement unit, and statistical information are provided where available from the original study.

Particular care was taken to distinguish microbial killing, loss of cultivability, reduction in biomass, inhibition of attachment, biofilm detachment, metabolic changes, and structural alterations. These outcomes were treated as biologically distinct and were not considered directly equivalent measures of antibiofilm efficacy. Quantitative effects were therefore interpreted in relation to the specific endpoint measured rather than compared solely on the magnitude of the reported reduction.

### 10.4. Limitations

This review has several methodological limitations. Studies were purposively selected rather than identified through exhaustive record-by-record systematic screening, which may introduce selection bias, including potential bias toward studies with more readily interpretable quantitative outcomes, and limits reproducibility. Only English-language publications indexed in PubMed/MEDLINE and Scopus were considered, while Web of Science and Embase were not searched. No formal risk-of-bias assessment was performed. The included studies should therefore not be interpreted as an exhaustive representation of all available evidence within each physicochemical category.

The predominantly in vitro and ex vivo evidence base also limits direct clinical translation. Experimental models were frequently single-species and only partially reproduced the complexity of clinically established biofilms and host–tissue interactions. Effective exposure conditions may not always be achievable or tolerable in vivo because of constraints related to penetration, treatment geometry, heating, or cytotoxicity. Animal and other in vivo studies were outside the predefined scope; consequently, the findings summarized here should not be interpreted as establishing in vivo therapeutic effectiveness or clinical benefit. The exclusion of dental and orthodontic studies also limits representation of the extensive oral-biofilm literature, particularly for aPDT.

Magnetic-field studies present additional limitations related to heterogeneity in experimental models, exposure conditions, assessment endpoints, and evidence vintage. Direct magnetic-field exposure was represented by fewer contemporary studies, whereas magnetothermal, magnetomechanical, and magnetically guided approaches were predominantly represented by recent work. Although the latter more frequently reported substantial quantitative antibiofilm effects among the selected studies, these findings should not be interpreted as evidence of superior efficacy across the literature as a whole.

## 11. Conclusions

Physicochemical approaches provide a diverse set of tools for the detection, characterization, prevention, and disruption of medically relevant biofilms. Electrical and electrochemical methods, ultrasound, light-based treatments, plasma and thermal approaches, nanomaterials, surface modification, and magnetic-field-based systems can influence biofilms through fundamentally different physical and biological mechanisms. At the same time, physicochemical sensing techniques increasingly enable longitudinal or spatially resolved assessment of biofilm development and treatment response. Their outputs, however, should not be regarded as direct substitutes for microbiological measurements, as electrical, mechanical, optical, metabolic, and structural signals reflect different properties of the biofilm and its interface with the surrounding material.

Across the approaches reviewed, treatment efficacy was strongly dependent on the microorganism, biofilm maturity and model, surface or material, exposure parameters, and assessment method. Inhibition of initial attachment, reduction in retained biomass, loss of cultivability, metabolic suppression, structural disruption, and biofilm detachment represent distinct outcomes and should not be interpreted interchangeably. Consequently, robust evaluation of physicochemical interventions requires complementary biological endpoints together with adequate characterization of the applied field, energy source, material, surface, or other relevant physicochemical parameters. Greater standardization of experimental reporting would substantially improve comparison between studies and help distinguish true biological differences from methodological variability.

This distinction is particularly important for magnetic-field-based approaches. Direct magnetic-field exposure, magnetothermal treatment, magnetomechanical actuation, and magnetically guided micro- or nanosystems represent mechanistically different strategies. Among the purposively selected studies, magnetothermal and magnetomechanical approaches more frequently reported substantial quantitative antibiofilm effects, whereas direct magnetic-field responses were more variable and less standardized. This comparison applies to the selected study set and should not be generalized to the literature as a whole.

Future studies should therefore incorporate rigorous magnetic dosimetry, appropriate field-, material-, temperature-, and actuation-matched controls, and complementary measurements of viability, biomass, architecture, metabolism, detachment, and regrowth. For magnetically actuated systems, assessment of effects on surrounding host cells and tissues will also be important for defining a meaningful therapeutic window.

Overall, physicochemical methods offer substantial opportunities both for improved biofilm monitoring and for interventions that complement conventional antimicrobial strategies. Their further development will depend less on demonstrating isolated reductions in individual biofilm endpoints and more on establishing reproducible mechanisms, standardized experimental conditions, clinically representative models, and concordance between complementary measures of biofilm response. Such methodological integration will be essential for translating promising physicochemical and magnetic-field-based approaches from experimental biofilm systems toward clinically applicable strategies.

## Figures and Tables

**Table 1:** Comparative overview of physicochemical approaches to medically relevant biofilms.

Approach	Principal Physicochemical Mechanism	Main Biofilm Application	Typical Endpoints	Major Strengths	Principal Interpretive Limitations
Electrical/electrochemical	electric fields, interfacial electrochemistry, generated antimicrobial species	monitoring/treatment	EIS, CFU, biomass	real-time monitoring; possible antibiotic potentiation	signal is platform-dependent; interrogation may affect biology
Ultrasound/acoustic	cavitation, shear, mechanical disruption, enhanced penetration	treatment/preanalytical recovery	CFU, biomass, detachment	strong physical disruption	detachment ≠ killing
Light/aPDT	ROS, photothermal effects	treatment	CFU, metabolism, biomass	spatial control; multimodal combinations	penetration and photosensitizer dependence
Plasma/thermal	RONS or heat	treatment	CFU, architecture, metabolism	large reductions possible	thermal/plasma dosimetry; host effects
Nanomaterials	material toxicity, ROS, ion release, matrix interactions	prevention/treatment	biomass, adhesion, CFU	multifunctionality	assay interference; formulation dependence
Surface modification	wettability, charge, topography, active coatings	prevention	adhesion, biomass, CFU	device relevance	mainly preventive rather than mature-biofilm eradication
Magnetic	direct field, heating, torque, remote actuation	treatment/guidance	CFU, biomass, architecture	remote/non-contact actuation	major mechanistic and dosimetric heterogeneity

RONS: reactive oxygen and nitrogen species.

**Table 2:** Interpretation of commonly used biofilm assessment endpoints.

Endpoint/Method	Primary Measures	Interpretive Limitations	Technical Limitations/Interferences	Recommended Complementary Assessment
CFU	Recoverable cultivable organisms	Total biomass, VBNC cells, matrix	Culture conditions, detection limit	Biomass/structural assay
Crystal violet	Retained biomass	Viability; cells vs. matrix	Dye/material interactions	CFU or viability assay
XTT/resazurin	Metabolic activity	Direct viable cell count	Nanoparticle/reagent interference	CFU
EIS	Biofilm–electrode physicochemical response	Direct viable burden	Platform/frequency dependence	CFU/biomass
QCM-D	Hydrated mass/viscoelasticity	Viability	Surface/platform dependence	Microbiological endpoint
OCT/confocal imaging	Architecture/thickness/spatial structure	Cultivability	Segmentation/imaging conditions	CFU/metabolism
Detached-cell counts	Released organisms	Killing vs. physical removal	Sampling/recovery dependent	Residual surface biomass + viability
Regrowth	Residual capacity for recovery	Initial mechanism of reduction	Follow-up conditions	Immediate viability/biomass

## Data Availability

No new data were created or analyzed in this study. Data sharing is not applicable to this article.

## Supplementary Materials

The following supporting information can be downloaded at https://www.mdpi.com/article/10.3390/antibiotics15100965/s1, Supplementary Table S1: Representative studies included in the structured narrative synthesis: experimental models, intervention conditions, endpoints, findings, and limitations.

## Abbreviations

The following abbreviations are used in this manuscript:

AMF	Alternating magnetic field
aPDT	Antimicrobial photodynamic therapy
CFUs	Colony-forming units
EIS	Electrochemical impedance spectroscopy
FICI	Fractional inhibitory concentration index
MRSA	Methicillin-resistant *Staphylococcus aureus*
MRSE	Methicillin-resistant *Staphylococcus epidermidis*
OCT	Optical coherence tomography
PDA	Polydopamine
PEG	Poly(ethylene glycol)
QCM-D	Quartz crystal microbalance with dissipation monitoring
RMF	Rotating magnetic field
SPE	Screen-printed electrode
